# Helium‐based cold atmospheric plasma‐induced reactive oxygen species‐mediated apoptotic pathway attenuated by platinum nanoparticles

**DOI:** 10.1111/jcmm.12880

**Published:** 2016-06-02

**Authors:** Paras Jawaid, Mati Ur Rehman, Qing Li Zhao, Keigo Takeda, Kenji Ishikawa, Masaru Hori, Tadamichi Shimizu, Takashi Kondo

**Affiliations:** ^1^ Department of Radiological SciencesGraduate School of Medicine and Pharmaceutical SciencesUniversity of ToyamaToyamaJapan; ^2^ Plasma nanotechnology Research CentreNagoya UniversityNagoyaJapan; ^3^ Department of DermatologyGraduate School of Medicine and Pharmaceutical SciencesUniversity of ToyamaToyamaJapan

**Keywords:** helium cold atmospheric plasma, platinum nanoparticles, antioxidants, apoptosis, reactive oxygen species

## Abstract

Plasma is generated by ionizing gas molecules. Helium (He)‐based cold atmospheric plasma (CAP) was generated using a high‐voltage power supply with low‐frequency excitation (60 Hz at 7 kV) and He flow at 2 l/min. Platinum nanoparticles (Pt‐NPs) are potent antioxidants due to their unique ability to scavenge superoxides and peroxides. These features make them useful for the protection against oxidative stress‐associated pathologies. Here, the effects of Pt‐NPs on He‐CAP‐induced apoptosis and the underlying mechanism were examined in human lymphoma U937 cells. Apoptosis was measured after cells were exposed to He‐CAP in the presence or absence of Pt‐NPs. The effects of combined treatment were determined by observing the changes in intracellular reactive oxygen species (ROS) and both mitochondrial and Fas dependent pathway. The results indicate that Pt‐NPs substantially scavenge He‐CAP‐induced superoxides and peroxides and inhibit all the pathways involved in apoptosis execution. This might be because of the SOD/catalase mimetic effects of Pt‐NPs. These results showed that the Pt‐NPs can induce He‐CAP desensitization in human lymphoma U937 cells.

## Introduction

Plasma medicine is a rapidly growing interdisciplinary field combining engineering, physics, biochemistry and life sciences 1. Plasma, which has been regarded as the ‘fourth state of matter’, is a partially neutral ionized gas, which contains a mixture of electrons, photons, atoms, positive and negative ions, radicals and various excited and non‐excited molecules 2. Plasma is classified into two categories on the basis of the temperature application, namely ‘thermal’ and ‘non‐thermal’ or ‘cold’ atmospheric plasma. In general, non‐thermal or cold atmospheric plasma (CAP) is produced by applying a high‐voltage electric field at low pressures and power. Theoretically, it has been mentioned that any gas can be used to generate CAP 3. However, intensive research has been focused on the use of helium and argon because these noble gases are monatomic and chemically inert, which results in the production of a stable plasma 4. The use of helium has advantages over that of argon, it induces ionization at lower voltages and generates more reactive oxygen species (ROS) 5.

CAP has been found to be highly effective for biological and medical purposes including cancer treatment 6, 7, 8, 9, 10, 11. Several studies have shown the efficacy of CAP for cancer treatment and suggest that non‐thermal plasma can induce apoptosis of cancer cells in a dose‐dependent manner 11. CAP plasma can generate ROS mainly superoxide, peroxide, oxygen and hydroxyl radicals. Therefore, mounting evidence suggests that the effects of CAP plasma are mainly mediated *via* generation of ROS 12 and lead to apoptosis 11, cellular necrosis 13, and senescence 14.

Here, we explore the effects of platinum nanoparticles (Pt‐NPs) on He‐CAP‐induced ROS generation and apoptosis. The medicinal use of platinum (Pt)‐based compounds has gained attention since the discovery of the antitumor activity of cis‐Diamminedichloroplatinum (cis‐platin; discovered in 1960 and approved for clinical use in 1978) 15. Pt‐NPs have also been shown to induce DNA damage and p53‐mediated growth arrest 16. Platinum‐based therapeutic drugs, notably cisplatin and carboplatin, have been exploited in chemotherapy to kill cancer cells 15. On the other hand, NPs of some noble metals, including platinum, function as reducing catalysts because of the large surface area 17. The large surface area of small particles can potentiate the catalytic activity of metals, whose colloidal forms contribute to efficient catalysis with high electron holding at the surface 17, 18, 19. In biological systems, this ability has been regarded as superoxide dismutase (SOD)/catalase mimetic activity, which could be useful for the prevention of a number of oxidative stress‐associated pathologies 20, 21. Therefore, it is quite evident that Pt‐NPs in a biological system can exert differential effects including cancer prevention and treatment.

To resolve this issue, this study was designed to determine the effects of Pt‐NPs on He‐CAP‐ induced apoptosis. The molecular mechanisms underlying the effect of Pt‐NPs on He‐CAP‐induced cell death were determined by analysing the changes in the markers of both intrinsic and extrinsic pathways. The changes in the He‐CAP‐induced ROS production were also monitored. The Pt‐NPs used in this study were capped with polyacrylate (PAA), which make them stable in their colloidal solution 19. These PAA‐capped Pt‐NPs have been reported to be superior to EUK‐8, a well known SOD/catalase mimetic 22; in addition, their *in vivo* activity has also been well‐established 21.

## Material and methods

### Preparation of Pt‐NPs

Pt‐NPs were prepared by the citrate reduction of H_2_PtC_l6_, in accordance with a previous report with minor modifications 17. Briefly, 43.8 ml H_2_O was poured into a 100 ml eggplant‐type flask and 4 ml of 16.6 mM H_2_PtC_l6_ was added. The reaction mixture in the flask was stirred at 100°C until reflux started. An 8.6 ml aliquot of 77.2 mM trisodium citrate dihydrate was injected into the reaction mixture and reflux was continued for additional 30 min. A change in the colour of the reaction mixture from light yellow to dark brown or dark red was observed, thus indicating the start of platinum reduction and nanoparticles formation. The reaction mixture was cooled to room temperature, 10 ml of 3.96 mg/ml pectin was added and the mixture was stirred for 1 hr. More pectin was added to improve the stability of the Pt‐NPs. The original molarity of platinum was 1 mM. To prepare the required concentration, Pt‐NPs were diluted with RPMI 1640 and DMEM containing 10% foetal bovine serum to a final concentration of 300 μM.

### Cell culture

Human myelomonocytic lymphoma U937, HeLa, HCT‐116, Molt‐4 and Jurkat‐T cell lines were obtained from Human Sciences Research Resource Bank (Japan Human Sciences Foundation, Tokyo, Japan). The U937, Molt‐4 and Jurkat‐T cells were grown in RPMI 1640 culture medium, HeLa and HCT‐116 cells were grown in DMEM supplemented with 10% heat‐inactivated foetal bovine serum (FBS) at 37°C in humidified air with 5% CO_2_.

### Cold atmospheric helium plasma irradiation system

A cold atmospheric plasma system (PN‐120TPG, NU Global, Nagoya, Japan) consisted of a gas flow controller, a voltage power supply and a hand‐piece of the plasma jet, constructing an inner micro‐hollow‐type electrode and an outer dielectric barrier electrode. The inner and outer diameter of dielectric tube was 1 and 2 mm respectively. A high‐voltage power with a frequency of 60 Hz and a peak‐to‐peak voltage of 7 kV was supplied to the two electrodes. Helium gas with a gas flow rate of 2 l/min. was applied in this study for the generation of a plasma jet. The line‐averaged electron density in the plasma source is approximately 2 × 10^15^ cm^−3^. The length of the plasma jet was approximately 20 mm in atmospheric ambient. The gas temperature of the plasma jet was below 350 K.

### He‐CAP treatment

U937, Molt‐4 and Jurkat‐T cells (0.5 × 10^6^) were cultured in a 24 well plate with 1 ml of RPMI1640, HeLa and HCT‐116 cells (0.5 × 10^6^) were cultured in a 24 well plate with 1 ml of DMEM. Cells were irradiated at a distance of 2 cm from the tip of plasma jet tube to the solution surface and were harvested at the indicated time periods.

### Cell viability

The trypan blue exclusion test was performed by mixing 50 μl of a cell suspension with an equal amount of 0.3% trypan blue solution (Sigma, St. Louis, MO, USA) in PBS. After 3 min. of incubation at room temperature, the numbers of stained and unstained cells were counted using a Burker Turk haemocytometer to estimate the number of non‐viable cells and viable cells respectively; at 24, 48 and 72 hrs post‐treatment.

### Cell counting assay

Cells were counted to determine the growth rate at 24, 48 and 72 hrs post‐treatment incubation. Samples of each treatment were collected and counted using a Burker Turk haemocytometer.

### Detection of apoptosis by using Annexin V‐FITC/PI staining

To determine early apoptosis and secondary necrosis, after the treatments, the cells were incubated at 37°C for 12, 18 and 24 hrs, collected, washed with cold PBS at 4°C and centrifuged at 100 × *g* for 3 min. The resulting pellet was mixed with the binding buffer of an Annexin V‐FITC kit fluorescein isothiocyanate (FITC)‐labelled annexin V. FITC‐labelled annexin V (5 μl) and propidium iodide PI (5 μl), (Immunotech, Marseille, France) were added to a 490 μl suspension and mixed gently. After incubation at 4°C for 20 min. in the dark, the cells were analysed by flow cytometry (Epics XL, Beckman‐Coulter, Miami, FL, USA).

### DNA fragmentation assay

For the detection of apoptosis, the percentage of DNA fragmentation was assessed 24 hrs post‐treatment using the method of Sellins and Cohen with minor modifications 23. Briefly, approximately 3 × 10^6^ cells were lysed using 200 μl of lysis buffer (10 mM Tris, 1 mM EDTA; and 0.2% Triton X‐100, pH 7.5) and centrifuged at 13,000 × *g* for 10 min. Subsequently, each DNA sample in the supernatant and the resulting pellet were precipitated in 25% trichloroacetic acid (TCA) at 4°C overnight and quantified using a diphenylamine reagent after hydrolysis in 5% TCA at 90°C for 20 min. The percentage of fragmented DNA in each sample was calculated as the amount of DNA in the supernatant divided by total DNA for that sample (supernatant plus pellet).

### Morphological detection of apoptosis

The morphological changes in the cells were examined by Giemsa staining. After 24 hrs of incubation at 37°C, the cells were harvested by centrifugation and washed in PBS. Then the cells were fixed in methanol and acetic acid (3:1) and spread on the glass slides. After drying, the cells were stained with 5% Giemsa solution (pH 6.8) for 15 min.

### Cell cycle analysis

For flow cytometry, cells were fixed with 70% ice cold ethanol, stored overnight at −20°C, and subsequently treated with 0.25 mg/ml RNase A (Nacalai Tesque, Kyoto, Japan) and 50 μg/ml PI to obtain the distribution of PI‐based cell cycle phases. The samples were finally run on an Epics XL flow cytometer (Beckman Coulter, Fullerton, CA, USA).

### Assessment of intracellular ROS generation by using DCFH and HE

Intracellular ROS generation in U937 cells was evaluated by flow cytometry using the fluorescence generated in the cells loaded with the sensitive fluorescent probes, a H_2_O_2_ sensitive dye, dichlorofluorescein diacetate (DCFH‐DA) (Molecular probes, Eugene, OR, USA). Hydroethidine (HE) (Molecular Probes) was used to determine superoxide generation O_2_^−^. The cells were pre‐incubated with Pt‐NPs at a dose of 300 μM for 3 and 6 hrs then exposed to He‐CAP for 4 min., immediately after post‐treatment; DCFH‐DA was added at a final concentration of 10 μM and HE was added at a final concentration of 5 μM. The fraction of fluorescence positive cells was measured by flow cytometry as the proportion of cells containing intracellular ROS.

### Measurement of mitochondrial transmembrane potential

After the treatments, the cells were incubated at 37°C for 24 hrs, collected, washed with PBS and centrifuged at 100 × *g* for 3 min. Then the cells were stained with 10 nM TMRM (Molecular Probes), for 15 min. at 37°C in 1 ml of PBS, followed by the immediate flow cytometry of red TMRM fluorescence.

### Measurement of [Ca^2+^]_i_

To monitor the effect of He‐CAP on intracellular calcium homeostasis, intracellular free Ca^2+^ was measured using the calcium probe Fluo‐3/AM (Dojindo Laboratories Co., Ltd., Kumamoto, Japan). Cells were treated with Pt‐NPs at 300 μM and He‐CAP for 4 min. After 18 hrs of incubation, the cells were harvested and then loaded with 5 μM Fluo‐3/AM for 30 min. at 37°C. Excess Fluo‐3/AM was removed by washing three times with PBS. The fluorescence intensity of free Ca^2+^ level was measured by flow cytometry.

### Western blot analysis

Cells were collected and washed with cold PBS. They were lysed at a density of 2.5 × 10^6^ cells/ml of RIPA buffer [50 mM Tris–HCl, 150 mM NaCl, 1% Nonidet P‐40 (v/v), 1% sodium deoxycholate, 0.05% SDS, 1 μg of each aprotinin, pepstatin and leupeptin and 1 mm phenylmethyl sulfonyl fluoride] for 20 min. Following a brief sonification, the lysates were centrifuged at 12,000×*g* for 10 min. at 4°C, and the protein content in the supernatant was measured using a Bio‐Rad protein assay kit (Bio‐Rad, Hercules, CA, USA). Protein lysates were denatured at 96°C for 5 min. after mixing with 2 μl SDS‐loading buffers, applied on an SDS–polyacrylamide gel for electrophoresis, and transferred to a nitrocellulose membrane. Western blot analysis was performed to detect caspase‐3, XIAP, Bid, Bax, Bcl‐2, Bcl‐xl and Fas expressions using specific antibodies. Antibodies were obtained from Cell Signaling technology (Danvers, MA, USA). Blots were then probed with either secondary horseradish peroxide (HRP)‐conjugated anti‐rabbit or antimouse IgG antibodies obtained from Cell Signaling. Band signals were visualized on a luminescent image analyser (LAS 4000, Fujifilm Co., Tokyo, Japan) by using chemiluminescence ECL detection reagents (Amersham Biosciences, Buckinghamshire, UK).

### Flow cytometric detection of Fas on cell surface

Cells (1 × 10^6^) were washed twice with ice cold PBS, resuspended in 20 μl of washing buffer containing 2.5 μg/ml of FITC‐conjugated anti‐Fas monoclonal antibody (clone:UB3, MBL, Nagoya, Japan) and incubated for 30 min. at room temperature. After staining the cells were washed again with ice cold PBS and re‐suspended in PBS. Data were analysed by using the flow cytometry.

### Measurement of caspase‐8 activity

To measure caspase‐8 activity, a FLICE/caspase‐8 colorimetric protease assay kit (MBL, Nagoya, Japan) was used according to the manufacturer's instructions. The assay was based on the spectrophotometric detection of chromophore p‐nitroanilide (pNA). The cells were harvested, lysed and the protein lysate was collected. The protein samples (100 μg) were mixed with 50 μl of 10 mM dithiothreitol (DTT) at a final concentration of 200 μM. The mixtures were then incubated at 37°C for 5 hrs and then the activity was measured at 400 nm using a spectrophotometer (Beckman Instruments Inc., Fullerton, CA, USA).

### Statistical analysis

The results are expressed as the mean ± standard error of the mean (SEM). All experiments were performed in triplicate. Significance was assessed by using unpaired Student's *t*‐test (two‐tailed) and differences were assumed significant for *P*‐values <0.05.

## Results

### Effects of He‐CAP on cell viability and apoptosis

**Figure 1:**
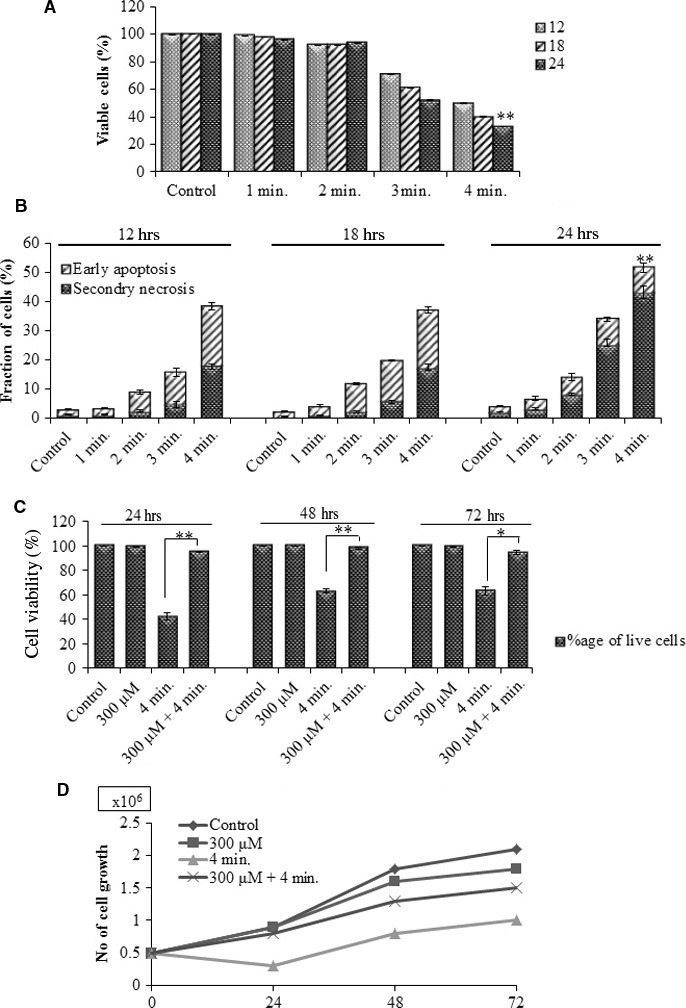
He‐CAP‐induced cell death in U937 cells. Cells were treated with He‐CAP at a dose of (1, 2, 3 and 4 min.). (**A**) Cell viability assessment and (**B**) annexin V‐FITC/PI assay were carried out at 12, 18 and 24 hrs after He‐CAP treatment. (**C**) Viability of cells in the presence or absence of Pt‐NPs. (**D**) Cell growth or cell proliferation. Cells were harvested after treatment with He‐CAP in the presence or absence of Pt‐NPs. The number of cells/ml was counted at 24, 48 and 72 hrs to assess proliferation. Data are presented as mean ± SD. *P < 0.05, **P < 0.005. Data shown are representative of five independent experiments. He‐CAP, Helium‐based cold atmospheric plasma.

Human lymphoma U937 cells were treated with He‐CAP for 1, 2, 3 and 4 min. and trypan blue exclusion dye assay was performed to assess the viability of cells at 12, 18 and 24 hrs. As shown in (Fig. 1A) dose and time‐dependent decreased in the viability of cells was observed in the cells treated with He‐CAP as compare to control. Furthermore, to determine the effects of He‐CAP on apoptosis, the cells were subjected to annexin V‐FITC and PI double staining. Flow cytometry revealed that He‐CAP treatment significantly increased the percentage of apoptotic cells in a dose and time‐dependent manner (Fig. 1B). As increased apoptosis was observed at He‐CAP treatment for 4 min., we used He‐CAP 4 min. exposure in the subsequent experiments.

### Effects of Pt‐NPs on He‐CAP‐induced apoptosis

To investigate whether Pt‐NPs have an ability to protect against apoptosis induced by He‐CAP, Pt‐NPs at a dose of 300 μM were used in this study based on our previous results 24 U937 cells were treated with He‐CAP for 4 min. in the presence or absence of Pt‐NPs at 300 μM, and the trypan blue exclusion dye was used to assess cell viability. As shown in (Fig. 1C) the percentage of dead cells was significantly decreased upon combined treatment at 24, 48 and 72 hrs compare to the cells treated with He‐CAP alone. Further, to confirm the growth of U937 cells, U937 cells were counted under a microscope using a haemocytometer at 24, 48 and 72 hrs after post‐treatment. Cell count in the combined treatment showed significantly increased growth of cells compare to He‐CAP treated cells (Fig. 1D).

**Figure 2:**
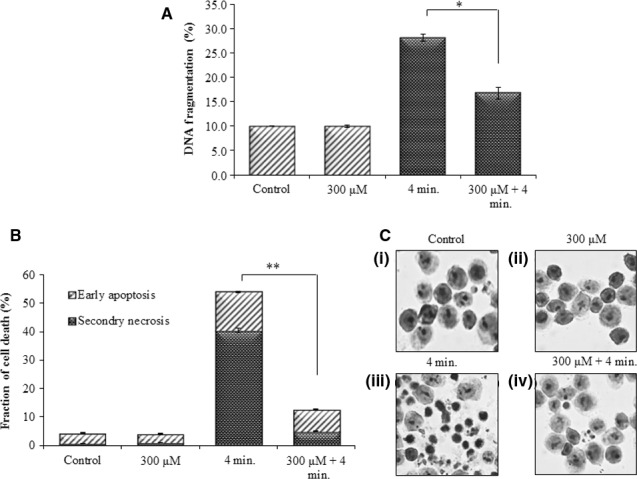
Effect of Pt‐NPs on apoptosis induced by He‐CAP in U937 cells. (**A**) DNA fragmentation assay (**B**) Percentages of early apoptotic and secondary necrotic cells were analysed 24 hrs after He‐CAP treatment with or without Pt‐NPs by flow cytometry. Data are presented as mean ± SD. *P < 0.05, **P < 0.005. Data shown are representative of five independent experiments. (**C**) Morphological features of apoptosis in U937 cells following treatment with He‐CAP in the presence or absence of Pt‐NPs; cells were harvested 24 hrs after treatment, signs of apoptotic features in response to He‐CAP were detected by Giemsa staining and then examined under a microscope at ×400 magnification. He‐CAP, Helium‐based cold atmospheric plasma.

U937 cells treated with He‐CAP resulted in a large percentage of apoptotic cell death as manifested by the increased in the percentage of DNA fragmentation, a hallmark of apoptosis which reaches up to 28.0 ± 2.0%. This increase in the DNA fragmentation % was significantly decreased, in the presence of Pt‐NPs at 300 μM (Fig. 2A).

Furthermore, to distinguish between the early and late‐phase of apoptosis, cells were subjected to annexin V‐FITC and PI double staining. The flow cytometry revealed that the percentage of early apoptotic cells decreased from 13.5 ± 1.0% following He‐CAP treatment to 7.0 ± 1.0% in the presence of Pt‐NPs at 300 μM. On the other hand the percentage of secondary necrotic U937 cells significantly decreased from 40.5 ± 3.0% to 5 ± 1.0% respectively (Fig. 2B). Moreover, giemsa staining showed that typical morphological changes related to apoptosis such as chromatin condensation and nuclear fragmentation were more prominent in the cells treated with He‐CAP alone [Fig. 2C (iii)]. However, these morphological changes were markedly decreased in the presence of Pt‐NPs. [Fig. 2C (iv)].

### Effects of Pt‐NPs on He‐CAP‐induced cell cycle distribution

U937 cells were treated with He‐CAP in the presence or absence of Pt‐NPs at 300 μM. The effects of combined treatment on cell cycle distribution are shown in (Table 1), the percentage of cells in the sub G1 was increased following He‐CAP treatment (24.0 ± 2.5). However, in the presence of Pt‐NPs the percentage of cells in sub G1 significantly decreased from 24.0 ± 2.5 to 9.8 ± 0.5. This increase in the percentage of sub G1 population was determined by a decrease in cells in the G1 and G2/M phases, which may be caused by apoptosis.

**Table 1:** Cell cycle analysis by flow cytometry. Data are presented as mean ± SD. Data shown are representative of five independent experiments

	Control	Pt‐NPs (300 μM)	He‐CAP (4 min.)	Pt‐NPs (300 μM) + He‐CAP (4 min.)
Sub G1	3.7 ± 0.2	3.8 ± 0.8	24 ± 0.4	9.8 ± 1.2^a^
G0/G1	41.3 ± 0.1	40.7 ± 1.0	30 ± 0.7	38.5 ± 0.6
S phase	33.4 ± 2.6	35 ± 0.6	30 ± 1.5	26.7 ± 0.5
G2/M	18.2 ± 0.7	17.7 ± 0.2	13 ± 1.4	21 ± 1.6

He‐CAP, Helium‐based cold atmospheric plasma; Pt‐NPs, platinum nanoparticles.

a Indicate statistical significance compared to He‐CAP.

### Effect of Pt‐NPs on He‐CAP‐induced ROS generation

**Figure 3:**
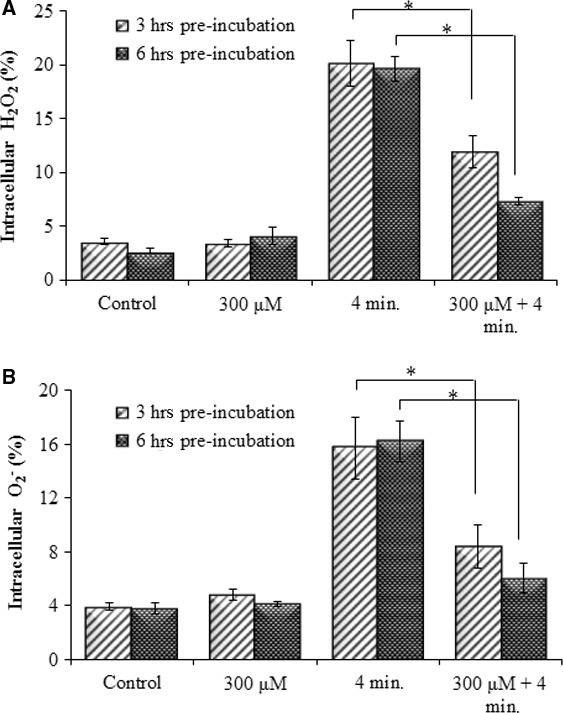
Effect of Pt‐NPs on He‐CAP‐induced ROS formation in U937 cells. U937 cells were treated with He‐CAP for (4 min.) in the presence or absence of Pt‐NPs. The percentage of cells with elevated level of ROS species were analysed by flow cytometry immediately after He‐CAP. (**A**) DCFH staining, (**B**) HE staining. Data are presented as the mean ± SD. *P < 0.05, as compared with He‐CAP treatment alone. Data shown are representative of five independent experiments. He‐CAP, Helium‐based cold atmospheric plasma; ROS, reactive oxygen species.

Reactive oxygen species generation plays an important role in He‐CAP‐induced apoptosis; we examined the effects of Pt‐NPs on He‐CAP‐induced ROS production in U937 cells. The effects of Pt‐NPs on He‐CAP‐induced ROS generation are plotted in (Fig. 3A and B). U937 cells were pre‐treated with Pt‐NPs at 300 μM for 3 and 6 hrs before He‐CAP treatment. Flow cytometry with DCFH‐DA and HE staining was used to detect certain species of intracellular ROS production in treated cells. A marked increase in the production of ROS was observed immediately after He‐CAP treatment in the cells. However, Pt‐NPs at 300 μM significantly reduced He‐CAP‐induced ROS production (*P* < 0.05 *vs* He‐CAP alone).

### Measurement of mitochondrial membrane damage

**Figure 4:**
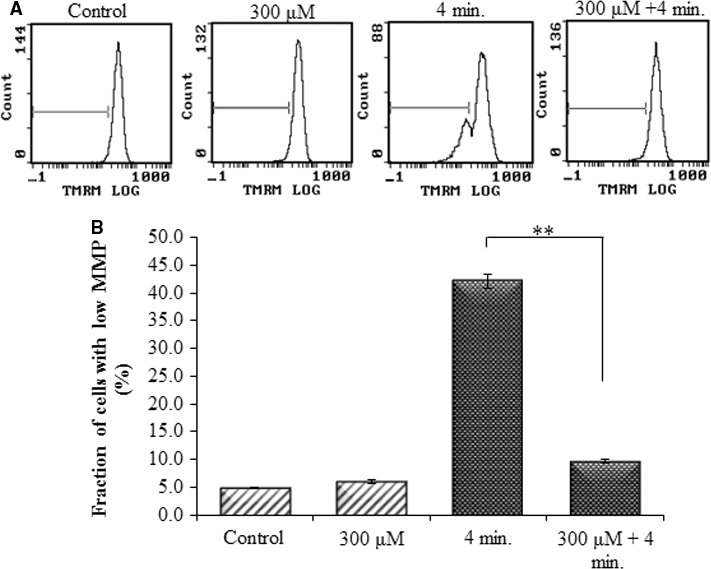
Effects of Pt‐NPs on He‐CAP‐induced MMP loss in U937 cells, as determined by flow cytometry using TMRM staining (**A**) Representative flow cytometric histogram of MMP loss. (**B**) Increased loss of MMP was observed in the cells treated with the He‐CAP alone which was decreased in the presence of Pt‐NPs. Data are presented as mean ± SD. **P < 0.005 as compared with He‐CAP treatment alone. Data shown are representative of five independent experiments. He‐CAP, Helium‐based cold atmospheric plasma; MMP, mitochondrial membrane potential.

To assess the involvement of the mitochondrial apoptotic pathway in the inhibition of He‐CAP‐induced apoptosis by the combined treatment, the effects of the treatments on mitochondrial membrane potential (MMP) were evaluated. Immediately after treatment with Pt‐NPs, the cells were exposed to He‐CAP treatment. It was found that the MMP loss (▵Ψm), which is the end point of apoptosis, was significantly decreased in the cells treated with Pt‐NPs (10.0 ± 0.3%) than the cells treated with He‐CAP alone (34.3 ± 1.5%) (Fig. 4A and B). The fraction of cells with low MMP percentage was analysed by flow cytometry using TMRM staining.

### Effect of Pt‐NPs on intracellular [Ca^2+^]_i_ levels

**Figure 5:**
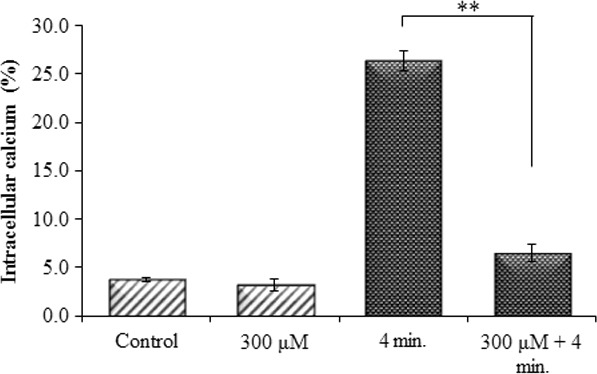
Effects of Pt‐NPs on He‐CAP‐induced [Ca^2+^]*i* levels. Cells were treated with He‐CAP for (4 min.) and then harvested 18 hrs after treatment. Cells were loaded with 5 μM calcium probe Fluo‐3/AM for 30 min., and [Ca^2+^]*i* level was measured by flow cytometry. Data are presented as mean ± SD. **P < 0.005, compared with the He‐CAP alone. Data shown are representative of five independent experiments. He‐CAP, Helium‐based cold atmospheric plasma.

Changes in [Ca^2+^]_i_ levels were assessed by flow cytometry. The results showed that intracellular [Ca^2+^]_i_ levels were increased by approximately 20‐fold to the basal levels following He‐CAP treatment alone. On the other hand, Pt‐NPs significantly suppressed the He‐CAP‐ induced increased [Ca^2+^]_i_ level (Fig. 5).

### Expression of apoptosis‐related proteins

**Figure 6:**
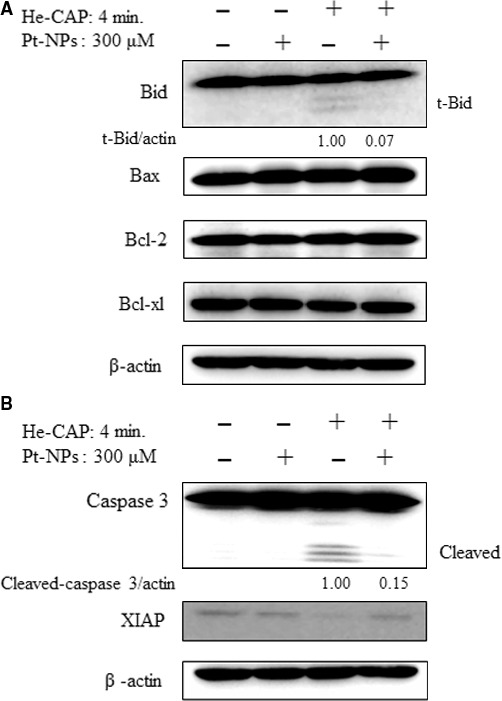
Assessment of apoptosis‐related proteins. Cells were treated with or without Pt‐NPs and, then harvested 18 hrs after helium‐based cold atmospheric plasma treatment. (**A**) Western blot analysis of Bcl‐2 family proteins (**B**) Changes in the expression levels of caspase‐3 and Xiap as detected by Western blot analysis. The t‐Bid and cleaved caspase‐3 signals were normalized to β‐actin and the relative ratios are shown below the band. Band density was evaluate by Image J software and expressed as fold change.

Western blot analysis revealed that the increased expression level of the pro‐apoptotic Bcl‐2 family protein, truncated Bid (tBid, an active form of Bid) was observed following He‐CAP treatment alone. However, in the presence of Pt‐NPs the expression level of tBid was markedly decreased. While, no significant changes in the expression levels of pro‐apoptotic Bax and anti‐apoptotic Bcl‐2, Bcl‐xl were observed after treatment either with He‐CAP alone or in combination with Pt‐NPs (Fig. 6A).

Caspases play a key role in the apoptosis and considered as the main executioner of apoptosis signalling pathways. The effects of combined treatment on the activation of caspase‐3 were evaluated. Following He‐CAP treatment, the expression level of the active form of caspase‐3 (cleaved caspase 3) was markedly increased. However, the combined He‐CAP treatment with Pt‐NPs strongly suppressed the expression of the caspase‐3 active form. Furthermore, to confirm the activation of the caspase cascade, the expression level of the x‐linked inhibitor of the apoptosis protein XIAP was determined by Western blot analysis. As XIAP has been known as the potent inhibitor of caspase‐3 and stop apoptotic cell death, it was found that following combined treatment, the XIAP expression level was higher than He‐CAP treatment alone (Fig. 6B).

### Effect of Pt‐NPs on He‐CAP‐induced FAS externalization and caspase‐8 activation

**Figure 7:**
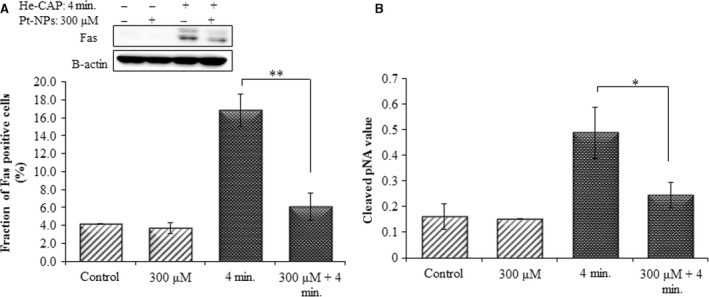
Effects of Pt‐NPs on He‐CAP‐induced Fas externalization and caspase‐8 activation. (**A**) Expression of Fas detected by Western blot analysis. Changes in the fraction of U937 cells with Fas externalization were analysed by flow cytometry using an anti‐Fas FITC‐conjugated antibody after treatment with He‐CAP (4 min.) for 18 hrs in the presence or absence of Pt‐NPs. (**B**) Caspase‐8 activity in U937 cells induced by the He‐CAP treatment with or without Pt‐NPs was also measured with a FLICE/caspase‐8 colorimetric protease kit. Data are presented as mean ± SD. *P < 0.05, **P < 0.005 compared with the He‐CAP alone. Data shown are representative of five independent experiments. He‐CAP, Helium‐based cold atmospheric plasma.

The Fas receptor is a death receptor on the surface of cells that leads to one of the apoptotic pathways, that is, the extrinsic pathway, through DISC assembly and subsequent caspase‐8 activation. The Fas externalization and caspase‐8 activity were detected in U937 cells at 18 hrs after He‐CAP treatment. The results showed that Pt‐NPs significantly suppressed He‐CAP‐induced Fas externalization and caspase‐8 activation (Fig. 7A and B).

### Effects of Pt‐NPs on He‐CAP‐induced apoptosis in other cancer cell lines

**Figure 8:**
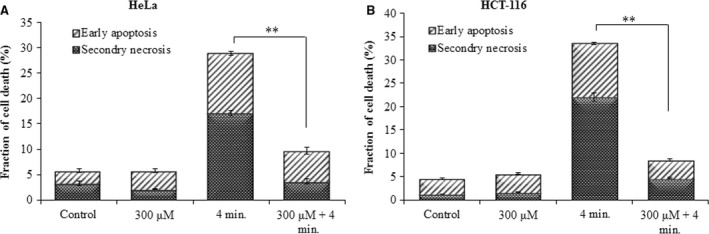
Effect of Pt‐NPs on He‐CAP‐induced apoptosis in other cell lines. Evaluation of early apoptosis and secondary necrosis by annexin V‐FITC/PI staining (**A**) HeLa cells (**B**) HCT‐116 cells. Data are presented as mean ± SD. **P < 0.005 compared with He‐CAP alone. Data shown are representative of five independent experiments. He‐CAP, Helium‐based cold atmospheric plasma.

The effects of Pt‐NPs in combination with He‐CAP were also determined in other cancer cell lines namely Molt‐4, Jurkat, HeLa and HCT‐116 cells. In HeLa and HCT‐116 cells, the percentages of early apoptosis and secondary necrotic cells after 24 hrs were significantly decreased in the presence of Pt‐NPs 300 μM than in He‐CAP treatment alone (Fig. 8A and B).

Similarly, in Molt‐4 and Jurkat cells PI staining 6 hrs after He‐CAP treatment showed a significant increase in the percentage of apoptotic cells in the absence of Pt‐NPs, which was suppressed in the presence of Pt‐NPs (Sup Figure 1A and 2A). In addition, trypan blue assay at 6 hrs also showed the protective effects of Pt‐NPs on He‐CAP‐induced apoptosis (Sup Figure 1B and 2B).

## Discussion

In this study, it was demonstrated that treatment with Pt‐NPs suppressed He‐CAP‐induced apoptosis. Therefore, the assumptions regarding the usefulness of Pt‐NPs in combination with He‐CAP seem to be true. It has been demonstrated that the electromagnetic, optical, and catalytic properties of noble‐metal nanocrystals are strongly influenced by their shape and size 25, 26. However, the toxicity of platinum‐based compounds is also well‐established 27. One such commonly used cytotoxic agent in cancer chemotherapy is cisplatin, which exerts its effects by interfering with transcription and other DNA‐mediated cellular functions 28. Further, it was reported that Pt‐NPs are capable of inducing DNA damage and p53‐mediated growth arrest 16. Considering, the facts mentioned above, one may speculate that the combined treatment of Pt‐NPs with He‐CAP may enhance the effect of He‐CAP on inducing apoptosis. However, our study showed that Pt‐NPs inhibit He‐CAP‐induced apoptosis. This discrepancy may be because of the fact that Pt‐NPs exert their antitumor and cytotoxic effects are through the lethal DNA damage caused by soluble Pt ion species, resulting in DNA strand breaks and the formation of Pt‐DNA complexes. Furthermore, it has also been known that the size of nanoparticles plays a crucial role and various sizes of Pt‐NPs (<20, <100, >100 nm) exert no influence on proliferation or growth inhibitory effects indicating that Pt‐NPs do not exhibit any serious cytotoxicity even at higher concentrations 16, 29, 30, 31.

Several studies, concerning the antioxidant effects of Pt‐NPs showed that Pt‐NPs can reverse the hyperthermia (HT) and X‐irradiation‐induced apoptosis through the suppression of all the involved micro‐molecular pathways particularly *via* suppression of intracellular ROS 24, 32, as well as protects mouse RAW24.7 macrophages from LPS‐induced inflammation by scavenging ROS 32. Inconsistent, the Pt‐NPs used in this study were believed to quench ROS, owing to their large surface area of smaller particles 17, 33.

Growing evidence indicates that CAP has the potential to be a safe anticancer therapy that can be used for selective killing of various cancer cells, such as breast, liver, ovarian and leukaemia 34, 35, 36, 37. It is well known that CAP‐induced cell death is mainly mediated by the generation of ROS, including^**.**^OH, H_2_O_2_ and O_2_^−^ 38. CAP‐induced ROS (or their reaction products) in the liquid phase, which diffuse through the plasma membrane or react with the plasma membrane to produce intracellular ROS through lipid peroxidation 39, 40, resulting in the release of growth factors in proliferating cells 41. Which ultimately caused damage to intracellular components, either promote or inhibit the intracellular signalling pathways 42, 43 and DNA damage in cancer cell lines 11, 12. It was reported that H_2_O_2_ and O_2_^−^ are the major species involved in the apoptosis and cell death induced by CAP. In this study, it was demonstrated that treatment with Pt‐NPs protects He‐CAP‐induced apoptosis in U937 cells, which is mainly because of the scavenging effect of Pt‐NPs on He‐CAP‐induced H_2_O_2_ and O_2_^−^ generation, suggesting the SOD/catalase mimetic activity of Pt‐NPs. These findings are consistent with the observation that treatment of cells with H_2_O_2_ and O_2_^−^ scavengers can reverse the biological effects of plasma 44. As He‐CAP‐induced extracellular ROS might be scavenged immediately in the presence of Pt‐NPs, thereby preventing its further interaction with the plasma membrane, resulting in the reversal of He‐CAP‐induced effects in U937 cells. Therefore, the role of Pt‐NPs or He‐CAP at the cell membrane level might exist and also the intracellular effects on the protein level may prevent cells from undergoing He‐CAP‐induced apoptosis.

ROS activate the intrinsic apoptotic pathway because of their interaction with proteins with the mitochondrial permeability transition complex 45. In addition, ROS specifically H_2_O_2_ has also been reported to induce apoptosis by Fas up‐regulation 46. Furthermore, FAS/TNF‐R1 can cause apoptosis *via* the direct recruitment of the caspase cascade or *via* the mitochondria by activating caspase‐8 and Bid 47, 48. The intrinsic pathway involves the disruption of the mitochondrial membrane, release of mitochondrial proteins including cytochrome‐c, increase in [Ca^2+^]_i_, activation of Bcl‐2 family members and p53 49. These two apoptotic pathways ultimately trigger the effector caspases which results in cellular shrinkage, DNA fragmentation and could be interconnected by the caspase‐8‐mediated cleavage of Bid, which triggers the activation of the mitochondrial pathway. In this study, it was demonstrated that the fraction of cells with decreased MMP were substantially increased following the He‐CAP treatment with the concurrent increases in [Ca^2+^]_i_ and activation of caspase‐3. These findings suggest the involvement of the caspase‐dependent mitochondrial pathway in the He‐CAP‐induced apoptosis in U937 cells. Moreover, the involvement of the extrinsic pathway was confirmed from the increased FAS and caspase‐8 activation following He‐CAP treatment, which might result from the changes in intracellular ROS generation. Furthermore, increased Bid activation was observed following He‐CAP treatment. Bid can be cleaved by caspase‐8 and the cleaved Bid as the carboxyl‐terminal fragment translocates to the mitochondria to induce the release of cytochrome‐c 50, 51.

Previously, in the HeLa cell line, the atmospheric‐pressure plasma jet induced apoptosis was mediated through the mitochondria‐dependent pathway *via* the generation of free radicals, without any involvement of death receptors 52. In contrast, our findings showed the involvement of both the intrinsic and extrinsic pathways and the Ca^2+^‐dependent pathway of apoptosis following He‐CAP treatment in U937 cells. This discrepancy may be because of the induction of different cellular pathways induced by CAP depending on the target and cell line used. Therefore, it can be assumed that the caspase‐8‐mediated activation of Bid also contributed in the mitochondrial pathway during apoptosis induced by He‐CAP treatment in U937 cells. However, in the presence of Pt‐NPs the suppression of both the extrinsic and intrinsic pathways was observed *via* the reduction in Fas expression level and restoration of the decreased MMP. Taken together, this study showed positive correlation between the ROS scavenging activity of Pt‐NPs and their anti‐apoptotic roles against He‐CAP‐induced apoptosis.

**Figure 9:**
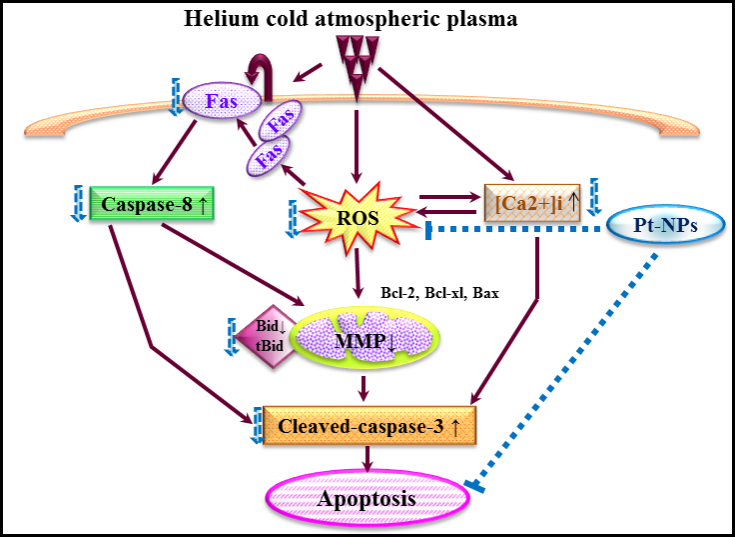
Graphical scheme of the pathways involved in the protective effects of Pt‐NPs against helium‐based cold atmospheric plasma‐induced apoptosis.

In conclusion, this study demonstrate that Pt‐NPs mainly scavenged He‐CAP‐induced ROS, resulting in the inhibition of the ROS‐mediated release of [Ca ^2+^]_i_ and activation of the Fas receptor, so that the activity of caspase‐3 and caspase‐8 was decreased, which prevent the loss of MMP and ultimately inhibit He‐CAP‐induced apoptosis (Fig. 9). It has been known that metal nanoparticles can induce differential effects depending on their physical properties. Therefore, care should be taken when selecting nanoparticles for future adjuvant therapy with CAP.

## Conflict of interest

The authors declare that they have no conflict of interests.
